# Oxidative DNA Damage: A Role in Altering Neuronal Function

**DOI:** 10.33696/signaling.3.079

**Published:** 2022

**Authors:** Adib Behrouzi, Mark R. Kelley, Jill C. Fehrenbacher

**Affiliations:** 1 Department of Pharmacology and Toxicology, Indiana University School of Medicine, Indianapolis, IN 46202, USA; 2 Indiana University Simon Comprehensive Cancer Center, Indiana University School of Medicine, Indianapolis, IN 46202, USA; 3 Herman B Wells Center for Pediatric Research, Department of Pediatrics, Indiana University School of Medicine, Indianapolis, IN 46202, USA; 4 Department of Biochemistry and Molecular Biology, Indiana University School of Medicine, Indianapolis, IN 46202, USA; 5 Stark Neuroscience Research Institute, Indiana University School of Medicine, Indianapolis, IN 46202, USA

**Keywords:** Oxidative stress, Oxidative DNA damage, Base excision repair, 8-oxoguanine DNA glycosylase-1, Apurinic/apyrimidinic endonuclease/redox effector factor 1, Chemotherapy-induced peripheral neuropathy, Inflammatory bowel disease, Alzheimer’s disease, Aging, Amyotrophic lateral sclerosis

## Abstract

A role for oxidative stress in the etiology of myriad neuropathologies is well accepted. However, the specific effects of oxidative DNA damage in the onset or promotion of neuronal dysfunction have been less studied. In our recent publication by Behrouzi *et al*. (Oxidative DNA Damage and Cisplatin Neurotoxicity Is Exacerbated by Inhibition of OGG1 Glycosylase Activity and APE1 Endonuclease Activity in Sensory Neurons), inhibition of enzymes that play a role in repairing oxidative DNA damage exacerbated neurotoxic effects of the chemotherapeutic agent, cisplatin. In this Commentary, we aim to expand on the contribution of oxidative DNA damage to other neuropathologies within the peripheral and central nervous systems, including irritable bowel disease, aging and Alzheimer’s disease, amyotrophic lateral sclerosis, and other neurodegenerative diseases. Consistently, clinical neuropathology and disease progression correlates with increases in oxidative DNA damage within clinical biopsies. Progress in animal models of these diseases has elucidated a causative role for oxidative DNA damage in disease progression, as dampening the DNA repair response exacerbates disease, whereas promoting DNA repair mitigates disease. Overall, this Commentary highlights the importance of expanding our studies on oxidative DNA damage in the nervous system, as enhancing oxidative DNA repair might prove to be a potential therapeutic target for the mitigation of neurodegeneration.

## Introduction

There are a wide range of neuropathologies in which oxidative and nitrooxidative stress play a predominant role in the etiology of the disease [1–3]. Extensive research efforts have focused on examining the effects of reactive oxygen and nitrogen species (ROS/RNS) on the production of oxidized proteins and lipid peroxidation within neurons [4–6]. Another byproduct of oxidative stress that has been understudied in neuroscience, however, is oxidative DNA damage and the role of that damage in affecting neuronal function. One potential reason for the paucity of neuroscience research in the area of oxidative DNA damage is the notion that DNA damage is less dangerous in fully differentiated neurons compared to other cell types, as neurons do not undergo cellular division. However, recent studies have highlighted the effects of oxidative DNA damage to increase transcription, decrease transcription, and alter signal transduction pathways [7–15].

**Figure 1:**
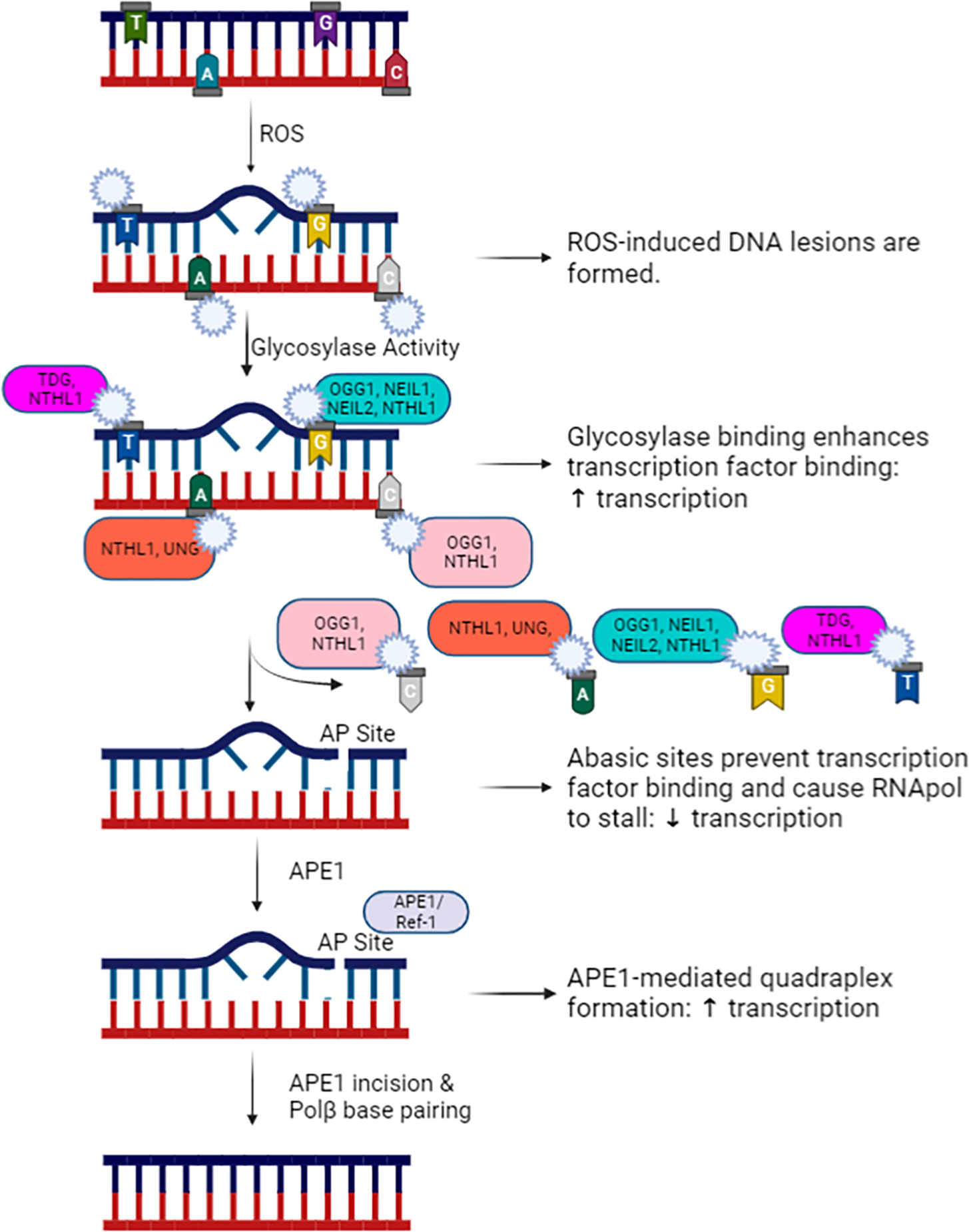
An overview of the base excision repair pathway with various base-specific glycosylases and stage-specific differential effects on transcription. Figure created in Biorender.com.

Reactive oxygen and nitrogen species are produced by normal cellular metabolism and excessive production is ameliorated by the activity of endogenous antioxidant mechanisms, which are robust in neurons [16]. Oftentimes, however, the antioxidant mechanisms can be overwhelmed and unmitigated ROS/RNS can cause oxidative DNA damage. Oxidative DNA damage is induced by the interaction between a highly reactive oxygen or nitrogen species and a DNA base, resulting in production of various base lesions. The most abundant oxidative DNA lesion is called 7,8-dihydro-8-oxo-2’-deoxyguanosine (8oxo-dG), which is formed at the 8^th^ carbon of the guanine base [17–19]. However, other lesions include 2,6-diamino-4-oxo-5-formamidopyrimidine (FapyG), which also forms on the guanine base; and 7,8-dihydro-8-oxoadenine (oxoA) on the adenine base. The relative abundance of these lesions highlights the importance of 8oxo-dG and FapyG; however, there is opportunity to explore less abundant lesions, such as oxoA and others, and to critically assess the relative incidence of these lesions in neurons relative to other cell types. As mentioned previously, oxidative lesions can cause altered DNA transcription and signal transduction [7–15] that can lead to functional impairment of cells. The base excision repair (BER) pathway is primarily responsible for the repair of oxidative DNA damage (See Figure 1 for a simplistic overview of the pathway). Cleavage of the damaged base is achieved by various glycosylases, depending on the lesion of interest. The primary glycosylase responsible for 8oxo-dG cleavage is 8-oxoguanine DNA glycosylase-1 (OGG1) [20]. There are other glycosylases that can recognize 8oxo-dG, including Nth Like DNA Glycosylase 1 (NTHL1) and Neilike DNA glycosylases 1 and 2 (NEIL1 and NEIL2), but they primarily recognize other pyrimidine lesions. Additional DNA glycosylases that recognize and cleave non-guanine lesions include Thymine-DNA glycosylase (TDG) and Uracil-DNA glycosylase (UNG). An abasic site is generated following removal of the base by the appropriate glycosylase, which is recognized and processed by AP endonuclease 1/Ref-1 (APE1/Ref-1). APE1/Ref-1 processes the ends on the DNA backbone, which allows DNA polymerase β (Polβ) to recognize and fill the abasic site with the correct base through template-directed synthesis [21]. In addition to the endonuclease function of APE1/Ref-1, the enzyme also has a redox function, regulating transcriptional activity via the reduction of a number of transcriptional factors [22]. Another enzyme that minimizes oxidative DNA damage is Nudix hydrolase 1 (also known as MTH1), which hydrolyzes oxidized bases within the nucleoside triphosphate pools to avoid incorporation of a damaged base into a nucleic acid [23,24]. The relative contributions of these different components of the base excision repair pathway to ward off deleterious effects of oxidative DNA damage are now being explored in different neuropathologies. Here we will briefly discuss our recent findings in sensory neurons exposed to DNA-damaging chemotherapeutics and explore the role of oxidative DNA damage in other peripheral and central nervous system pathologies.

## Peripheral Neuropathies

**Figure 2:**
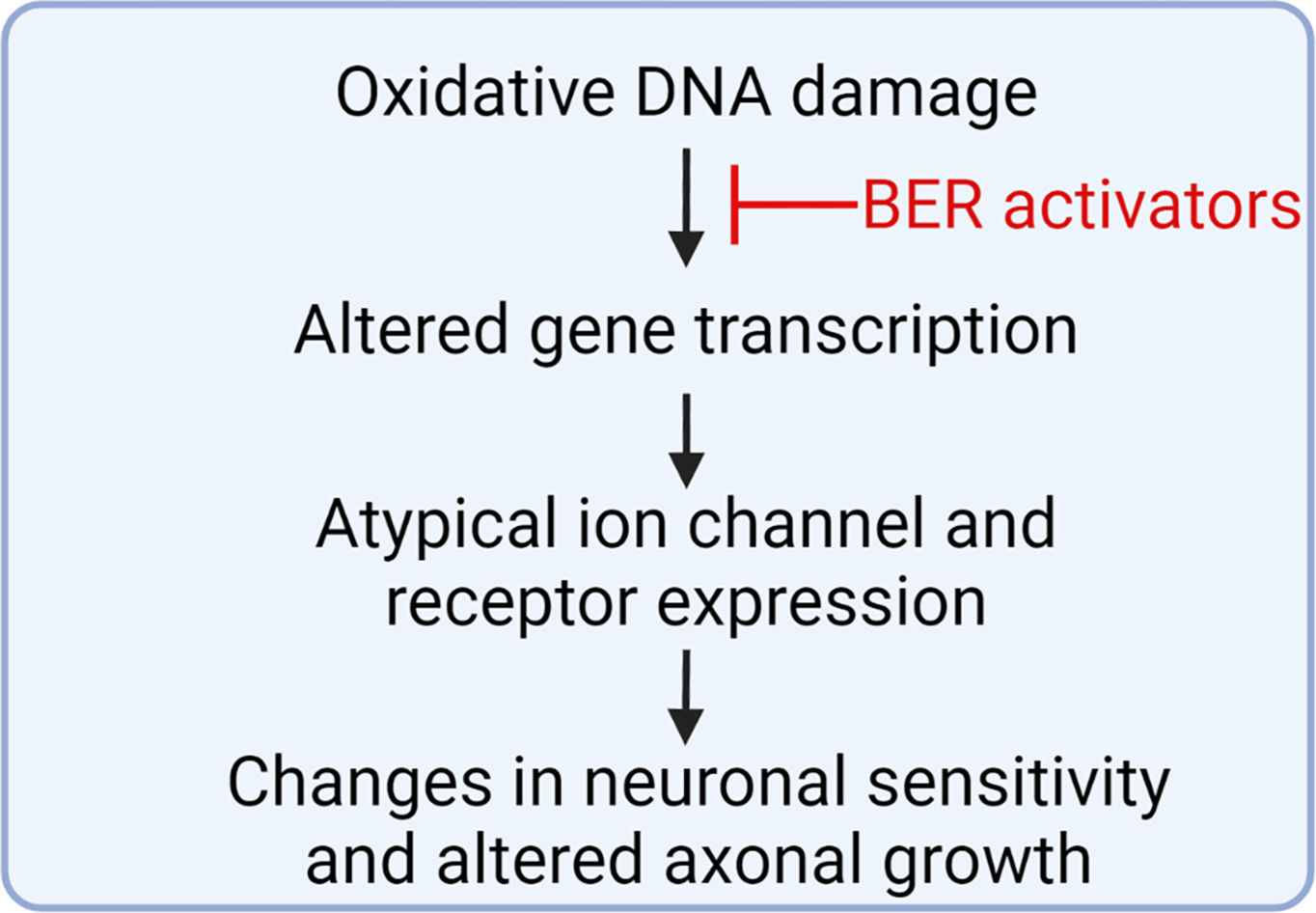
A proposed role for how enhancing DNA repair in sensory neurons could prevent or reverse chemotherapy-induced peripheral neuropathy. Figure created in Biorender.com.

Breakthroughs in chemotherapeutic treatments of cancer patients have enhanced survival of cancer patients over the past few decades, however these life-saving drugs can also cause adverse effects. Chemotherapy-induced peripheral neuropathy (CIPN) is an adverse effect of cancer treatment that can diminish the quality of life for survivors and, in severe cases, limit further administration of chemotherapeutic treatment due to severe neuropathic pain. Unfortunately, there are few available treatments for the pain associated with CIPN due to an incomplete understanding of how chemotherapeutics alter neuronal function [25]. Our laboratory group has postulated that oxidative DNA damage within sensory neurons mediates long-term transcriptional and signal transduction changes in sensory neurons that underlie the altered function of the neurons. We previously published that enhancing the BER pathway for DNA repair via increasing the activity of APE1/Ref-1 was neuroprotective against the effects of cisplatin in cultures of primary sensory neurons [26–28]. Recently, we expanded those studies to examine the role of the glycosylase that removes 8oxo-dG lesions, OGG1, as different stages of the BER pathway can alter transcription in distinctive ways (see Figure 1). We found that pharmacological inhibition of either OGG1 or APE1/Ref-1 increases 8-oxodG levels in neuronal cultures, even in the absence of any stressor, confirming that the BER pathway plays a large role in maintaining low levels of oxidative DNA damage in sensory neurons. Functionally, inhibition of the BER enzymes exacerbated the effects of cisplatin, causing augmented desensitization of neuropeptide release from sensory neurons. These data support a role for oxidative DNA damage, and specifically 8oxo-dG formation, as a mediator of cisplatin-induced neurotoxicity in sensory neurons. Future studies will further this line of questioning by elucidating the differential effects of OGG1 and APE1/Ref-1 modulation on neuronal transcriptional changes induced by cisplatin (see Figure 2). In addition to oxidative DNA damage, the platin drugs also elicit bulky DNA platin adducts, which contribute to functional deficits within sensory neurons [29,30] and are primarily repaired by the nucleotide excision repair pathway. Our recent study demonstrated that inhibition of OGG1 and APE1 enhanced the burden of adduct lesions induced by cisplatin, which supports previous findings by our group and others that elements of the BER pathway interact with the nucleotide excision repair pathway [31,32]. Thus, enhancing the repair of oxidative lesions could restore neuronal function through the removal of platin adducts. There are multiple non-platin chemotherapeutics that have been reported to induce oxidative stress [33–35], therefore the relative importance of oxidative DNA damage to mediate CIPN may extend beyond the known DNA damage inducers, cisplatin and oxaliplatin. Despite the protective effects of enhancing BER-mediated DNA repair in sensory neurons, it is possible that increasing BER activity could compromise the tumor cytotoxicity of anticancer treatments. However, studies with APX3330, a small-molecule therapeutic which has been shown to bind to APE1/Ref-1 and increase DNA repair activity in neurons to provide neuroprotection against cisplatin [26] have demonstrated that APX3330 does not compromise the cytotoxicity of cisplatin in non-small cell lung or bladder cancer cells [36,37]. In fact, combination treatment of the cancer cells with APX3330 and cisplatin enhanced the cytotoxicity and decreased invasiveness in bladder cancer and lung cancer cells, respectively [36,37]. These studies support the notion that therapeutics can be designed to selectively target oxidative DNA repair in neurons. Ideally, future studies will be conducted in tumor-bearing animals to assess the effects of putative therapeutics on anticancer efficacy. Oxidative stress is a hallmark of multiple other neuropathies, including diabetic neuropathy [38,39] and HIV treatment-induced neuropathy [40], suggesting a putative role for oxidative DNA damage in mediating these neuropathies as well. Furthermore, enhancing the DNA repair activity of APE1 has been proven to be neuroprotective in sensory neurons after insults caused by ionizing radiation and inflammatory stimuli, which also produce oxidative DNA damage [27,41].

## Irritable Bowel Disease

Oxidative stress also contributes to tissue damage in patients with Irritable Bowel Disease (IBD). Mucosal biopsies from patients with IBD have enhanced reactive oxygen intermediates, diminished endogenous antioxidants, and increased oxidative DNA damage, as measured by 8oxo-dG [42]. The specific role of oxidative DNA damage in the pathophysiology of IBD was recently assessed in an animal model of spontaneous chronic colitis [43]. In this study, the investigators examined the effects of enhancing the DNA repair activity, while simultaneously diminishing the redox activity of APE1/Ref-1 via the pharmacological administration of APX3330. APX3330 administration reversed gross histological changes to the distal colon, diminished leukocyte infiltration into the colon, and restored gastrointestinal motility in the animal model of colitis. Upon further examination, reversal of these symptoms was associated with an increased survival of myenteric nerves and glial cells with diminished superoxide production and 8oxo-dG formation in the myenteric plexus [43]. These data suggest a prominent role for colonic oxidative stress to induce transcriptional activity and oxidative DNA damage within the enteric nervous system and highlights APE1/Ref-1 as a potential therapeutic target for patients with IBD.

While neuropathies and IBD are largely a result of dysfunction within peripheral sensory neurons, oxidative DNA damage may mediate pathology of many diseases and the following sections highlight research demonstrating a role for oxidative DNA damage in central nervous system diseases.

## Aging and Alzheimer’s Disease

Increasing levels of oxidative stress and subsequent accumulation of DNA damage is a hallmark of aging and has been shown to underlie age-related and Alzheimer’s Disease-associated cognitive impairment [44,45]. Further, exacerbation of DNA damage via suppression or dysfunction of DNA damage repair proteins can accelerate age-related cognitive decline in rodents and humans [46,47], supporting a specific role for oxidative DNA damage in the etiology of aging. These results have been replicated in an animal model, whereby the activity of OGG1 is experimentally reduced. Suppression of OGG1 activity results in an increase in age-induced 8oxo-dG levels in mouse brains with a concomitant decline in cognitive function [48]. Similarly, the same authors explored the effects of compromised OGG1 activity in a 5XFAD mouse model of Alzheimer’s disease (AD) and found an increase in 8oxo-dG in hippocampal neurons and further deficits in cognition compared to 5XFAD mice with normal OGG1 activity levels [48]. Similar findings were described by Oka et al., who demonstrated that knockout of OGG1 and the nucleoside hydrolase, MTH1, which work collectively to diminish the burden of 8oxo-dG, exacerbated the accumulation of 8oxo-dG in brain microglia of triple-transgenic AD model mice (3xTg-AD-H) and promoted degeneration of the hippocampus [49]. Furthermore, the loss of MTH1 and OGG1 activity resulted in an exacerbation of cognitive impairment in the AD mice [49]. Clinically, oxidative DNA damage is validated, as 8oxo-dG levels are more prevalent in AD patient brains [1,50] and serum [51] compared to age-matched controls. This increase in oxidative DNA damage suggests increased production of ROS in AD patients that overwhelm repair mechanisms, a disease-related dysfunction or diminution of endogenous antioxidant or DNA damage repair activity, or some combination of the two. Altogether, these publications support the notion that oxidative DNA damage plays a key role in the development or progression of AD, thus further research on ways to enhance the repair of oxidative DNA damage is warranted to identify novel putative therapeutics for AD.

## Amyotrophic Lateral Sclerosis

Amyotrophic lateral sclerosis is a neurodegenerative disease of large motor neurons and oxidative stress is presumed to also contribute to the pathogenesis of the disease [52]. ALS patients exhibit elevated levels of 8oxo-dG in upper (motor cortex) and lower (spinal cord) motor neurons compared to age-matched controls [53–56] and motor neurons with prominent nuclear 8oxo-dG staining exhibit markers of cellular death/dying [56]. Interestingly, the genes for APE1/Ref-1 and OGG1 are hypomethylated in motor cortex and spinal cord motor neurons from ALS patients, suggesting continued expression of the repair proteins and the propensity for DNA repair, thus enhancing the activity of these proteins might be a viable therapeutic strategy for ALS. Indeed, in an animal model of spinal motor nerve degeneration via sciatic nerve avulsion where there is an increase in 8oxo-dG [57], overexpression of APE1/Ref-1 or OGG1 to enhance DNA repair and diminish the DNA damage response attenuates neuronal cell loss in the spinal cord [58].

### Other Neurodegenerative Diseases

Correlations between an increase in oxidative DNA damage and clinical pathology have also been established for Parkinson’s disease (PD), with increases in 8oxo-dG levels and increased levels of DNA repair enzymes in the substantia nigra of PD patients [59,60]. Similar increases in oxidative DNA damage have been observed in Huntington’s Disease (HD) patient samples and in brain from mouse models of HD [61,62]. An elegant study has demonstrated that huntingtin plays a role in engaging the BER pathway in response to oxidative stress and is recruited to sites of oxidative DNA damage, but that mutant huntingtin introduces deficiencies in BER, leading to the accumulation of excess oxidative DNA damage [63].

## Conclusions

The repair of oxidative DNA damage through engagement of the BER pathway in post-mitotic cells, such as neurons, is critical because the cells do not have robust alternative strategies to repair the damage, such as replication proofreading and mismatch repair [64]. As reviewed herein, there is strong evidence that oxidative DNA damage correlates with neuropathologies and moderate evidence that DNA damage contributes to the onset or progression of some diseases in both the peripheral and central nervous system. Behrouzi et al. (2022) built upon an existing body of work examining the role of oxidative DNA damage in CIPN; however, further work is needed to fully understand how oxidative DNA damage alters transcriptional profiles to alter neuronal function. Similarly, further work in other neurodegenerative diseases is necessary to identify a causal role for oxidative DNA damage and explore therapeutics that could promote DNA repair and potentially lead to breakthroughs in clinical patient care.
